# A survey of gastrointestinal parasites in dogs illegally entering the UK (2015–2017)

**DOI:** 10.1002/vro2.54

**Published:** 2023-01-11

**Authors:** Margaret A. Fisher, Beth Rees, Colin Capner, Susie Pritchard, Peter A. Holdsworth, Ronan A. Fitzgerald

**Affiliations:** ^1^ Ridgeway Research Ltd Gloucestershire UK; ^2^ Heathrow Animal Reception Centre Hounslow UK; ^3^ PAH Consultancy Ltd Canberra, Australian Capital Territory Australia; ^4^ Bayer Plc, Animal Health Reading UK

## Abstract

**Background:**

This study involving non‐compliant, seized dogs entering the UK surveyed endoparasites detected in faecal samples. A focus was placed on taeniid infection as the detection of these tapeworms acts as a marker for failure of effective tapeworm treatment.

**Methods:**

Individual faecal samples taken from 65 dogs over a 24‐month period were examined for helminth eggs, for protozoal oocysts and cysts, using a centrifugal flotation technique. Any sample presenting positive results for taeniid eggs had residual faeces examined using polymerase chain reaction to aid speciation of the tapeworm eggs. Additionally, a Baermann technique was used to assess faeces for lungworm larvae.

**Results:**

Patent endoparasite infection was detected in 27.7% of dog faecal samples. No sample was positive for lungworm larvae. Five dogs were co‐infected with *Isospora* spp. and *Toxocara canis*. One dog sample was detected with taeniid eggs, identified as *Taenia serialis*.

**Conclusions:**

The taeniid‐positive dog indicated that appropriate tapeworm treatment may not have occurred, reinforcing the risk to the UK of illegally imported dogs potentially introducing *Echinococcus multilocularis* infection.

## INTRODUCTION

The Pet Travel Scheme (PETS) has regulated the legal importation of non‐commercial companion animals into the UK since 2000, replacing a mandatory 6‐month quarantine period. Although quarantine was designed to prevent the entry of rabies, it was recognised that its existence helped to prevent the entry of other infections as well. When PETS was introduced, the scheme required that imported animals had veterinary‐certified treatments documented within a passport for rabies, tapeworm and ticks; the tapeworm treatment aimed to reduce the risk of *Echinococcus multilocularis*, a major zoonotic threat, entering the UK.

The requirement for tapeworm treatment for dogs, as well as rabies vaccinations for dogs and cats, was maintained under the 2012 adjustments to PETS, which imposed a cost and management hurdle for individuals seeking to import companion animals. Nonetheless, it has been very successful, considering that the number of dogs entering the UK is increasing year on year, with 85,786 dogs in 2011 and 307,263 dogs in 2019 as cited.^1^

Non‐compliant importation of animals may occur inadvertently, but also intentionally to bypass controls. An absence of mandatory checks at entry means the scale of non‐compliant imports is difficult to measure and the risk such animals pose, hard to assess. Although reasons for non‐compliance may vary, this type of entry may represent a route for *E. multilocularis* because tapeworm treatment may not have occurred. It has been estimated that there was a 98% probability that, in the absence of praziquantel treatment, one dog in 10,000 would enter the UK carrying *E. multilocularis*.^2^

This study aimed to survey endoparasite, particularly taeniid, infection in dogs that had been seized following non‐compliant entry. Taeniid eggs act as a marker for failure of effective tapeworm treatment. The methodology for sample examination was therefore chosen to maximise the likelihood of identification of tapeworm infection, if present. The survey also provided an opportunity to identify the presence of other parasite species that could be exotic or non‐endemic to the UK.

## MATERIALS AND METHODS

**FIGURE 1:**
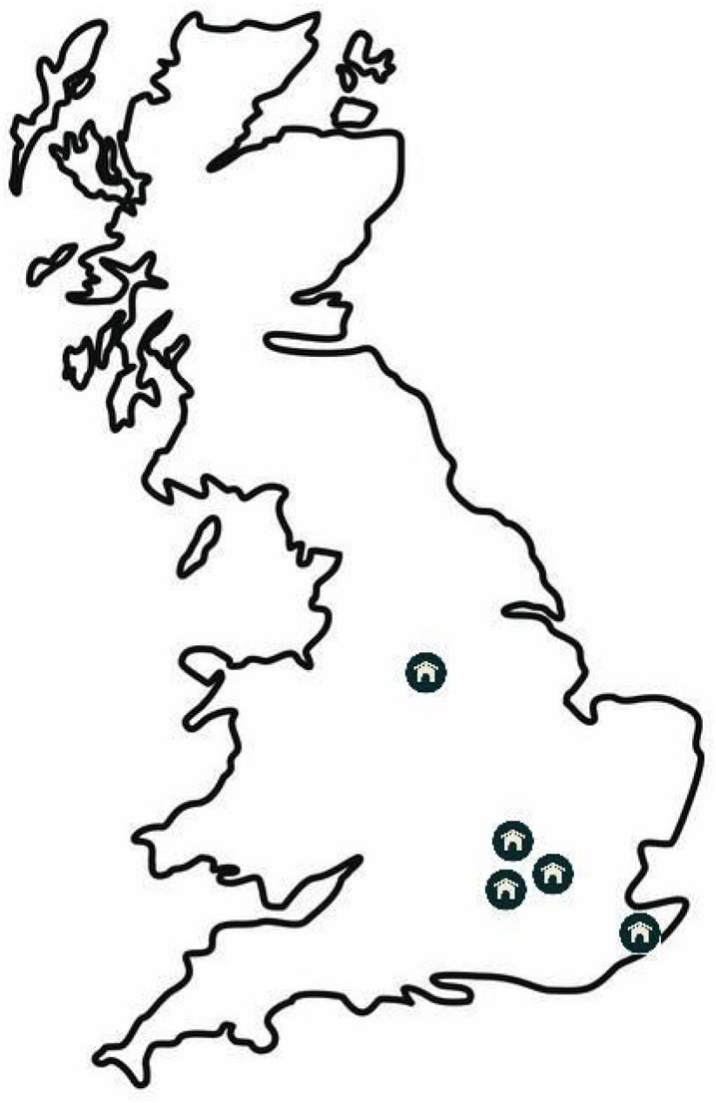
Map of Great Britain showing the approximate location of the kennels where samples were collected

Quarantine kennels and organisations responsible for holding or intercepting non‐compliant dogs were enrolled into the study (Figure 1) by staff at Heathrow Animal Reception Centre, UK. Relatively few kennels within the UK can accept animals that have been potentially illegally imported as they remain subject to quarantine regulations. Faecal samples were sought from any dog that had been imported, but failed to meet the regulations. As much detail as available about individual or group‐housed dogs (signalment; country of origin; sample date; recent anthelmintic dosing; presence of ectoparasites; diet; group‐ or sole‐housed) was recorded. Not all details were available for every animal, which was an inherent problem for the animals sampled.

Fresh faecal material was collected from the kennel or run floor and placed into provided containers. The pots were ascribed a unique identifier for the animal(s) concerned and the date of collection. Samples, together with data capture forms, were posted to Ridgeway Research, where they were processed. Samples were examined within a level 2 biosecurity facility for helminth eggs and protozoal oocysts or cysts indicating patent infections. A centrifugal flotation technique with Breza's solution was used as the flotation fluid (specific gravity of at least 1.3)^3^ in order to maximise the recovery of cestode eggs that require a higher specific gravity than nematode eggs.

Where taeniid eggs were found, 2–4 g of residual faecal matter from that individual dog was submitted to the University of Zurich for polymerase chain reaction examination to speciate the eggs recovered.

Separately, where there was sufficient faecal material, up to 10 g of faeces was placed in a Baermann apparatus and the resulting fluid examined for lungworm larvae.

Data were double entered and validated by means of an algorithm, and run within Microsoft Excel (Richmond, VA, USA), which compares and highlights differences between two spreadsheets. Any errors were checked against the original data and corrected. Data were summarised appropriately.

## RESULTS

Sixty‐five samples were received and processed over a period of 24 months, between 2015 and 2017. Samples were received from five quarantine establishments located in Figure 1, together with small numbers of samples from other organisations.

The dogs had been imported from a range of countries/regions, which are summarised in Table 1. The countries of departure varied considerably in terms of geography; prominent were Eastern European states and the USA. The majority of animals appeared to have been seized because of incorrect documentation or unspecified treatment prior to entry.

**TABLE 1:** Country or region of origin for dogs tested within the study (total 65)

Country or region	Number of dogs
Poland	9
USA	9
Hungary	8
Romania	8
Czech Republic	7
Lithuania	3
Moldova	3
Eastern Europe	2
Slovakia	2
Morocco	2
Qatar	2
Unknown	3
Egypt, Mexico, Slovenia, South Africa, Switzerland, Tanzania and Turkey	1 each

The age, sex and breed characteristics of the dogs are summarised in Tables 2 and 3. In all, 46.2% of dogs tested were female and 46.1% were male, with information on sex absent for the remaining dogs. Most dogs (66.2%) were aged under 6 months, while 4.6% were of unknown age. Of the animals sampled, 18 of 65 (27.7%) dogs were identified as having a patent endoparasite infection. The parasite species identified are listed in Table 4. Of 50 samples examined by Baermann, none was positive for lungworm larvae.

**TABLE 2:** Age and sex of dogs from which samples were obtained (total 65)

Sex	Age grouping	Number of dogs tested
Female	0–6 months	23
	6 months–1 year	1
	1–3 years	2
	3 years and above	4
Male	0–6 months	20
	6 months – 1 year	1
	1–3 years	3
	3 years and above	6
Undetermined	0–6 months	1
	1–3 years	1
Female	Undetermined	2
Male	Undetermined	1

**TABLE 3:** Breed of dogs from which samples were obtained (*n* = 65)

Breed	Number of dogs tested
French bulldog	9
Dachshund or Miniature Dachshund	8
Labrador	4
Cairn terrier	2
Maltese terrier	5
Cavalier King Charles or cross	2
Caen Corso	2
Chihuahua or cross	6
Cocker spaniel	2
Pug	3
Terrier X	2
Crossbred	4
Unknown	8
Beagle, Beauceron, Brussels Griffin, Dobermann, German shepherd dog, Pharoah hound, Samoyed, Yorkshire terrier	1 each

**TABLE 4:** Endoparasitic species recovered from illegally imported dogs

Endoparasite species	Number of positive samples	Percentage within test population (*n* = 65)
*Isospora* spp.	8	12.3
*Toxascaris leonina*	1	1.5
*Toxocara canis*	12	18.5
*Taenia serialis*	1	1.5

Five of the dogs tested were co‐infected with *Isospora* spp. and *Toxocara canis*.

One dog of undeclared age was identified to have *Taenia* spp. eggs within the faeces examined. The dog had been imported from Poland. The sequences from two samples taken matched two *Taenia serialis* sequences—using BLAST and sequence analysis with Clustal W in BioEdit and aligning with GenBank sequences—as given below:

9512976 3541179
UK_dog_Sample 1
ATATCTGGTTTAATATTATTGTTGAATAATATAAGTTTGTGTAATTTTATTAGTTAAGCCAAGTCTATGTGCTGCTTATAGAAGTATTCATGCGTTACTTTAATAAAGTTTTAGTTGTAAGCACTATTATATTTAGGACTTAAAAGTAATGTTAAATTAGTTTGTTAATGTGAAATAAGTTTAGCTCATGTACACACCGC
9512977 3541180
UK_dog_Sample 2
ACATTACTTTTAAGTCCTAAATATAATAGTGCTTACAACTAAAACTTTATTAAAGTAACGCATGAATACTTCTATAAGCAGCACATAGACTTGGCTTAACTAATAAAATTACACAAACTTATATTATTCAACAATAATATTAAACCAGATATACACCAACATAATAAAAGTAAATTAATAGGCGGAACATCCTTTACACCACACCTTCCCCTAAAAAGANTC

## DISCUSSION

The PETS was introduced in the UK in 2000 to facilitate international travel of companion animals within participating countries.^4^ In order to travel and not be subject to quarantine regulations, animals must possess a valid passport and receive specified treatments before entry into the UK. In this study, animals entering the UK were seized which originated from countries of departure that included both participants and non‐participants in PETS. Most were seized as a result of either lack of appropriate documentation or failures in completing the requirements for specified treatments (such as rabies vaccination). Animals that were deemed to infringe the regulations may be subject to quarantine, which involves kennelling in an approved establishment for 4 months or until travel requirements have been met.^5^

Access to illegally (or non‐compliant) imported dogs is problematic and studies have been limited. Access during quarantine is limited and the illegal import of dogs is covert and only seized animals offer the opportunity to be studied. Furthermore, there is an impetus to deworm seized animals at an early time point so as to prevent unnecessary exposure of staff to potentially zoonotic disease. This study was hence conducted on an opportunistic basis, was small and non‐systematic, even so we contend that it provides an insight into the parasite burdens of dogs that have been identified as non‐compliant and seized.

The preponderance (66%) of dogs aged less than 6 months travelling from Hungary and Romania would be consistent with investigations by the Dogs Trust (www.dogstrust.org.uk/)^6^ that revealed a significant incidence of puppy smuggling into the UK and particularly identified puppies travelling from Hungary and Serbia as being part of this trade. One further route from Spain was estimated to illegally import 20,000 puppies into the UK from a single supplier in a year.

There is still a requirement under PETS for animals to be treated with praziquantel or equivalent with proven efficacy against *E*. *multilocularis*. The treatment must have been given no less than 24 h and no more than 120 h (5 days) before entering the UK.^6^ Many of the dogs in this survey originated from Eastern European countries, where there is a substantial reservoir of *E. multilocularis* infection in red foxes. A survey estimated the prevalence more than 10% of foxes in the Czech Republic, Estonia, France, Germany, Latvia, Lithuania, Poland, Slovakia, Liechtenstein and Switzerland; while Austria, Belgium, Croatia, Hungary, Italy, the Netherlands, Romania and the Ukraine were estimated to have a prevalence more than 1% to less than 10%.^7^ There is increased awareness of an endemic region of *E. multilocularis* in northern USA and southern Canada.^8^

The tapeworms recovered from a dog sample were species that are endemic to the UK; however, for dogs entering the UK that should have been subject to praziquantel treatment—which exhibits extremely high efficacy against adult and larval cestodes^9^—it would be expected that no tapeworm should be found. It should be noted that routine checks are not made for the presence of parasites in dogs that are formally compliant. The presence of any tapeworm within this population of dogs suggests that gaps exist within the strategies for biosecurity at the UK border and that the potential exists for parasitic zoonotic infections to enter. Given the small sample size of this survey, it is disturbing that one dog was found to be carrying a taeniid infection and suggests that further work in this area is merited.

The presence of *T. canis* and *Isospora* spp. is consistent with a population of predominantly young dogs and puppies. *Toxocara canis* is a zoonotic parasite and is endemic in the UK. In this survey, 18.5% of dogs were found to be positive for *T. canis*, which is higher than the prevalence among the UK population of dogs^10^ and would also tend to reflect the presence of the parasite in the originating country as well as potential lack of anthelmintic use in the dogs prior to travel.

This small‐scale study identified one non‐compliant dog entering the UK with a patent taeniid infection. This indicates that such dogs may not have received effective tapeworm treatment and thus pose a risk of carrying and introducing more pathogenic tapeworms such as *E. multilocularis* infection. Risks of puppies entering the UK to fulfil market needs and bringing with them substantial parasite burdens require monitoring and control.

## AUTHOR CONTRIBUTIONS

Margaret A. Fisher, Peter A. Holdsworth and Ronan A. Fitzgerald devised the study and study design. Susie Pritchard oversaw sample collection. Beth Rees processed samples. All authors contributed to authorship, particularly Colin Capner and Peter A. Holdsworth. All authors approved the final version. This was consistent with ICMJE guidelines.

## CONFLICTS OF INTEREST

The authors declare they have no conflicts of interest.

## ETHICS STATEMENT

As the study did not involve any procedures on the dogs themselves no animal use approval was needed. Appropriate approval was obtained for collection of all samples in this study.

## Data Availability

The data that support the findings of this study are archived in Ridgeway Research and are available from the corresponding author upon reasonable request.
